# Lower uncertainty bounds of diffraction-based nanoparticle sizes

**DOI:** 10.1107/S1600576722002564

**Published:** 2022-04-29

**Authors:** İsmail Cevdet Noyan, Hande Öztürk

**Affiliations:** a Department of Applied Physics and Applied Mathematics, SEAS, Columbia University, 500 W 120th Street, New York, NY 10027, USA; b Department of Mechanical Engineering, Özyeğin University, Çekmeköy, İstanbul 34794, Turkey

**Keywords:** diffraction, particle size determination, Scherrer approach, integral breadth, Fourier analysis

## Abstract

A self-consistent analysis is reported of traditional diffraction-based particle size determination techniques applied to synthetic diffraction profiles generated with the Patterson approach. The results show that dimensions obtained from traditional techniques utilizing peak fitting or Fourier analysis for single-crystal nanoparticles have best-case error bounds of around 5%. For arbitrarily shaped particles, lower error magnitudes are possible only if the zeroes of the thickness fringes are used. The errors for sizes obtained by integral-breadth- and Fourier-decomposition-based techniques depend on the shape of the diffracting domains. In the case of integral-breadth analysis, crystal shapes which scatter more intensity into the central peak of the rocking curve have lower size errors. For Fourier-decomposition analysis, crystals which have non-uniform distributions of chord lengths exhibit nonlinearities in the initial ranges of the normalized Fourier cosine coefficient versus column length (|*A*_*L*_| versus *L*) plots, even when the entire rocking curve is used in the decomposition. It is recommended that, in routine analysis, all domain size determination techniques should be applied to all reflections in a diffraction pattern. If there is significant divergence among these results, the ‘average particle size(s)’ obtained might not be reliable.

## Abstract 2

The errors for particle sizes obtained by integral-breadth- and Fourier-decomposition-based techniques depend on the shape of the diffracting domains.

## 1. Introduction

Diffraction-peak-shape analysis techniques for determining crystallite size (also termed domain size) are very popular, possess a massive literature base and, in some cases, have NIST Standard Reference Materials (SRMs) (Cline *et al.*, 2020 ▸). However, the reliability, accuracy, precision and usefulness of the dimensions obtained from these techniques are still the subject of vigorous scientific debate. Some of the issues raised are common to all particle size determination methods. First, commonly used terms such as particle size, average particle size, particle size distribution *etc.* depend on the technique used for their determination and are not well defined for arbitrary particle shapes or characterization techniques (Matyi *et al.*, 1987 ▸). Second, different experimental techniques used for size characterization are sensitive to different parts of a given particle size distribution and can yield vastly different values for the ‘average’ size of the same assembly of particles. Thus, while none of these values are ‘wrong’, none, individually, can adequately describe the size of a particle of arbitrary shape, or an aggregate of such particles of different sizes. Further, such differences usually become more pronounced if, in addition to a size distribution, there is a distribution of particle shapes within the aggregate. In such cases even computed distributions of size within the aggregates might be technique dependent.

Disagreements are also encountered when one compares crystallite sizes obtained from different diffraction formulations. For unstrained single crystals scattering in the kinematic regime, the crystallite size obtained from formulations based on the Scherrer approach is the maximum real-space length^1^ of the coherently scattering domains along the scattering wavevector, 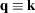 (Patterson, 1939*a* ▸,*b* ▸; Williamson & Hall, 1953 ▸). In contrast, Fourier analysis techniques of line shapes from such samples yield the average chord length along **k** (Guinier, 1994 ▸). For a given line profile, whether this average chord length obtained from Fourier methods is equal to the maximum crystal dimension obtained from the Scherrer approach depends on the shape of the crystal.

If there are additional sources of broadening in the diffraction line profile, as well as contributions from neighbouring peaks and the background intensity profile, obtaining an accurate and useful crystallite size from diffraction line profile analysis becomes more laborious and more error prone. This may be one of the reasons for the many conflicting statements in the literature about the efficacy of various formulations. For example, while some authors warn against the use (abuse) of the Scherrer equation (Matyi *et al.*, 1987 ▸; Palosz *et al.*, 2010 ▸; Leoni, 2019 ▸), others have concluded that it is a reliable technique, especially if strain broadening is negligible and lower-order peaks are used (Kaszkur, 2006 ▸; Ying *et al.*, 2009 ▸; Dorofeev *et al.*, 2012 ▸). Some authors discount the use of diffraction techniques altogether for specific applications. Tomaszewski (2013 ▸) asserted that critical grain sizes for size-induced phase transitions cannot be determined using diffraction methods, stating that ‘none of the known methods give the correct value for the crystallite size of nanocrystals! It is only possible to talk about the range of values.’

Another factor which complicates the comparison of crystal sizes obtained from various diffraction formulations is the lack of rigorous uncertainty values. Many experimental parameters influence the peak profile, some in a highly nonlinear manner, and the propagation of errors for some formulations is quite complicated. Currently there are no rigorous formalisms which can compute, from first principles, the full uncertainty range associated with crystallite sizes determined by diffraction methods (Young *et al.*, 1967 ▸).

In this work we have investigated the lower error bound (minimum uncertainty) associated with the accuracy and trueness (closeness between the average of an infinite number of replicate measured quantity values and a reference quantity value; Noyan *et al.*, 2020 ▸) of average crystal sizes obtained from the most popular diffraction formulations. For these computations we utilized the Patterson equations (Patterson, 1939*a* ▸,*b* ▸) to generate stand-alone diffraction peaks for various reflections of perfect Au crystallites with thin-film, cylindrical and spherical geometries. These intensity data are exact, with no counting uncertainty; thus, the peaks are exactly reproducible with infinite precision. These peak profiles were analysed for the average ‘particle size’ using the Scherrer equation (both FWHM and integral-breadth modes) and Fourier-decomposition approaches. Also, since the Patterson equations are based on exact geometric shapes, and the computed peaks are free from all confounding effects (such as limited angular range, background intensity, peak overlap and asymmetry), we expected this approach to provide the best achievable accuracy, and hence the minimum error bound, for particle sizes obtained by diffraction techniques.

## 2. Theory and simulations

We first reprise the 1939 Patterson formulation of the Scherrer equation (Patterson, 1939*a* ▸,*b* ▸) and compare its predictions of particle size with those of the Fourier analysis of line shapes (even though most of this material is available in the literature, it is scattered across many publications which are, mostly, somewhat terse treatments).

The Patterson formulation considers only kinematic diffraction which neglects multi-wave scattering and assumes that the energy diverted into the diffracted beam is negligible. Under these assumptions, for a plane wave 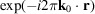 incident on a crystal, the wave 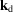 scattered by an atom at position 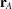 will have a phase difference 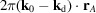 from the wave (with the same wavevector) scattered by an atom at the origin. The total diffracted wave amplitude in the direction of 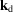 is the sum of the waves scattered by all the atoms in the crystal. Since X-rays are scattered by the core electrons surrounding the atoms, it is more accurate to consider the electron-density distribution 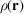 in the crystal rather than the atomic positions,

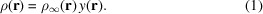

Here, 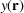 is the envelope function of the sample crystal, which is equal to 1 inside the crystal boundary and zero outside, and 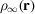 is the electron-density distribution of a triply periodic infinite crystal,

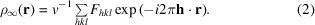


*v* and 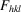 are the volume and structure factor, respectively, of the unit cell of the crystal, and 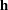 is the reciprocal-space vector.

The total diffracted wave amplitude is the sum of diffracted waves from all electrons over the entire crystal, taking into account the proper phase differences. This can be written as

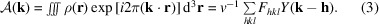

Here, 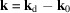 is the wavevector, and 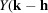 ), termed the shape function, 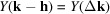 , is the Fourier transform of the sample envelope function 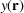 ,

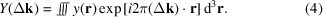




Equation (3) indicates that the shape of the Bragg peak in angular space is determined only by the real-space shape of the diffracting crystal. The type and chemistry of the unit cell modulate the amplitude at a given reciprocal-space position (or diffraction angle) mainly through the structure factor 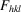 .

If the crystal is infinitely large in all directions, equation (3) yields Bragg’s law; in this case 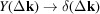 and finite amplitude (and thus intensity) will only be observed at the exact Bragg condition where 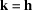 .

**Figure 1:**
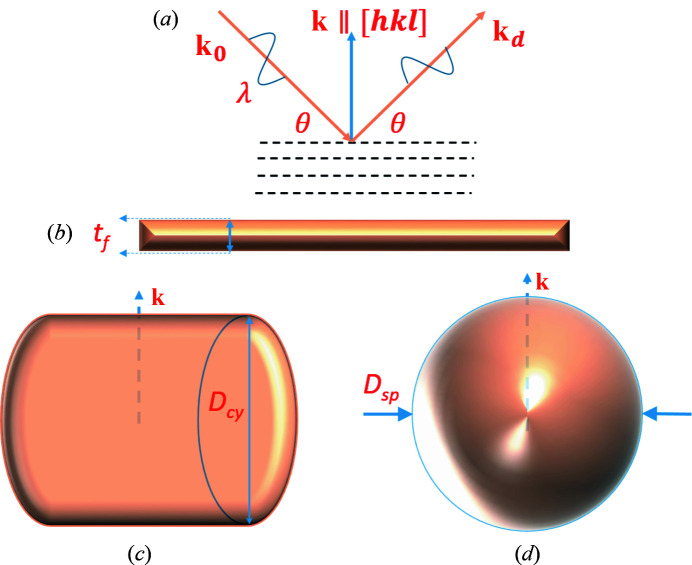
(*a*) The symmetric radial scan geometry for kinematically diffracting single crystals, with extra definitions for (*b*) a semi-infinite thin film, (*c*) a cylindrical shape and (*d*) a spherical shape.

For finite-sized crystals the shape of the diffraction peak, *i.e.* the angular distribution of scattered intensity around the Bragg angle, must be obtained by evaluating the triple integral in equation (4). Patterson and many others have computed 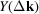 for many regular geometric shapes. In this study we will only consider three simple cases where the diffracting crystal is (i) a thin film, (ii) a cylinder and (iii) a sphere, respectively. The corresponding geometries are sketched in Fig. 1 ▸.

### 2.1. Diffraction from a single-crystal thin-film slab

We consider a radial scan from a perfect single-crystal slab of (real-space) thickness *t*_f_ and infinite in-plane area, where the diffracting (*hkl*) planes are parallel to the surface of the film with their normal [*hkl*] coincident with the normal to the film surface [Figs. 1 ▸(*a*) and 1 ▸(*b*)]. In this case evaluation of equation (4) yields the normalized cardinal sine (sinc) function,

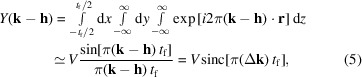

where *V* is the volume within the single-crystal thin film illuminated by the X-ray beam. By substituting this expression into equation (3) we obtain the amplitude at a given 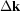 . The corresponding diffracted intensity along 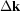 is obtained by multiplying the amplitude by its conjugate. Expressing 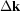 in terms of deviation from the Bragg angle, 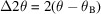 , we obtain

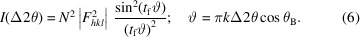

**Figure 2:**
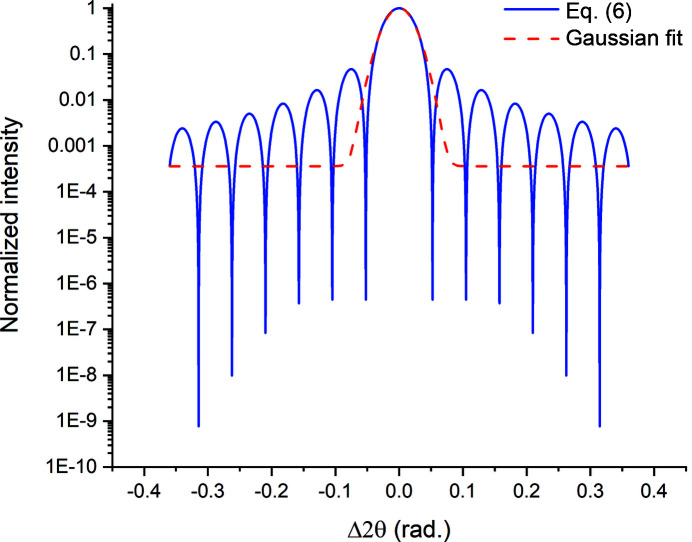
The variation in diffracted intensity with angular position around the Bragg position, Δ2θ = 0, for a radial scan of the 111 reflection from a hypothetical single-crystal Au thin film, 5 nm thick, illuminated with Cr *K*α radiation [blue line, equation (6)]. A Gaussian function fitted to the central peak is also shown (red dashed line).

Here, *N* is the number of unit cells along 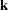 and *k* = 1/λ. Equation (6) shows that the shape of the (kinematic) diffraction peak from a perfect single-crystal thin film illuminated by a plane wave is described by the squared normalized sinc function^2^ 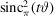 = 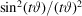 . When the thickness *t*_f_ approaches infinity, the intensity will be zero at all Δ2θ except Δ2θ = 0. For a finite thickness, the 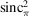 function will have a primary peak with its maximum intensity (*I*_max_ = 1) at Δ2θ = 0, which is bracketed by symmetric subsidiary peaks (thickness fringes) of diminishing intensity with increasing |Δ2θ|. An example pattern computed for a 5 nm thick Au film using Cr *K*α radiation is shown in Fig. 2 ▸. One can use three techniques to obtain the film thickness from this peak profile using equation (6):

(i) The periodicity of the thickness fringe zeroes, *T*, is related to the film thickness^3^ by

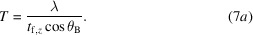




(ii) The half-maximum intensities of the diffraction peak will be reached when 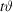 = ±1.39. Thus, the FWHM β of the primary peak will given by

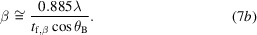

This is the Scherrer equation for FWHM, with the Scherrer constant *C* = 0.885.

(iii) The integral breadth β_I_ of the 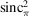 function is related to the film thickness by

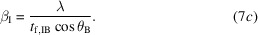

This is the integral-breadth form of the Scherrer equation with a Scherrer constant of unity.

While the forms of equations (7*a*)[Disp-formula fd7a]–(7*c*)[Disp-formula fd7b] [Disp-formula fd7c] are very similar, their efficacies in computing the film thickness are not. As we showed previously using high-resolution diffraction from silicon-on-insulator films (Ying *et al.*, 2009 ▸), equation (7*a*)[Disp-formula fd7a] is the easiest to use, and the most accurate, for single-crystal thin films since it does not utilize a ‘shape-dependent’ constant and the zeroes of the thickness fringes can be determined very precisely when high-resolution XRD systems on high-intensity sources are utilized. The FWHM form of the Scherrer equation requires the Scherrer constant for the particular geometry. Also, the FWHM obtained from an approximate function, such as a Gaussian, fitted to 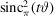 introduces a small error in β and hence in *t*_f,β_.

Equation (7*c*)[Disp-formula fd7c] also does not require a Scherrer constant. However, computation of the integral breadth β_I_ is non-trivial. Fitting only a portion of the 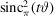 profile with an approximate function, such as a Gaussian (dashed line in Fig. 2 ▸), will cause errors in the computed film thickness *t*_f,IB_ since (i) the tails of the Gaussian function do not fit the tails of the central peak of the 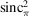 function and (ii) fitting only the central peak underestimates the integrated intensity of the radial scan; this would result in a film thickness *t*_f,IB_ larger than its actual value.

**Figure 3:**
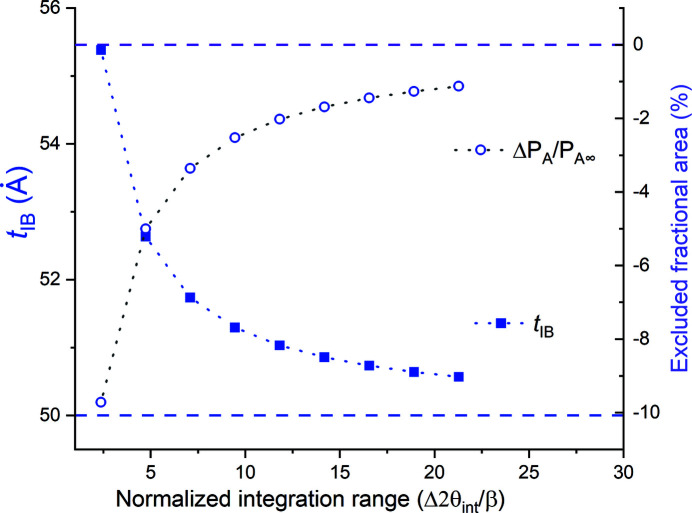
The variation in *t*_IB_ values with the angular integration range used for determining the integral breadth β_I_ in Fig. 2. The fractional excluded peak area, Δ*P*_A_ [equation (9)], for each such range is also shown. For convenience the integration ranges are expressed as ratios of the FWHM β of the central peak, (Δ2θ_*H*_ − Δ2θ_*L*_)/β.

To investigate this effect we computed β_I_ by evaluating the integral

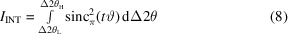

over various 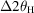 – 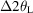 ranges in Fig. 2 ▸. For ease of interpretation we chose these ranges to include the central peak plus identical numbers of satellite peaks, bounded by their respective zeroes, on each side. In Fig. 3 ▸ we plot the *t*_f,IB_ values corresponding to these different integration ranges, starting with the range for just the central peak and ending with the range with the central peak plus eight satellite peaks on each side. In this figure we also include the fractional excluded peak area Δ*P*_A_ for each such range,

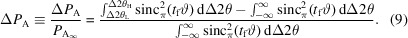

If just the integral breadth of the central peak is used to compute the film thickness, *t*_f,IB_ is ∼10% larger than the actual film thickness, since the area of the central peak is ∼10% smaller than the true total integrated intensity, 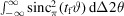 . As the angular range of integration increases, *I*_INT_ and *t*_f,IB_ asymptotically approach their ideal values; analysis of the range containing the central peak plus four satellites at each side (sixth point from the left) yields ∼2% deviation.

**Figure 4:**
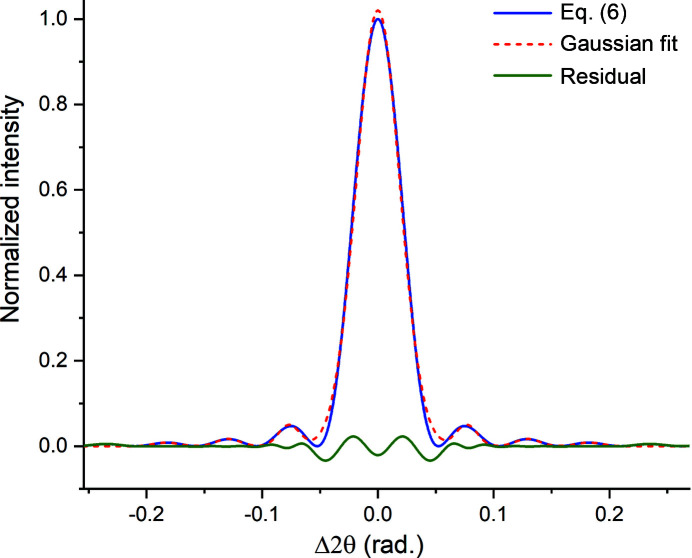
Seven Gaussian peaks fitted simultaneously to the diffraction peak shown in Fig. 2. The FWHM is insensitive to the number of satellites included in the fit.

To investigate this issue further we fitted multiple Gaussians to the profile shown in Fig. 2 ▸. An example with seven peaks (central peak plus three satellites on each side) is shown in Fig. 4 ▸. The *t*_f,IB_ values obtained using the sum of the integral intensities of all fitted peaks are summarized in Table 1 ▸. For ease of comparison the corresponding *t*_f,IB_ values from the integration of equivalent ranges of the 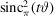 profile are also listed. We observe reasonable agreement between the two approaches.

Table 1 ▸ also shows that the film thickness values obtained from the FWHM form of the Scherrer equation, equation (7*b*)[Disp-formula fd7b], do not change significantly with increasing number of fringe peaks fitted simultaneously with the central peak. All of these values are within 5% of the actual (ideal^4^) thickness. As shown in the last row of Table 1 ▸, one obtains the best accuracy when using the zeroes of the thickness fringes [equation (7*a*)[Disp-formula fd7a]] to compute the film thickness *t*_f_.

**Figure 5:**
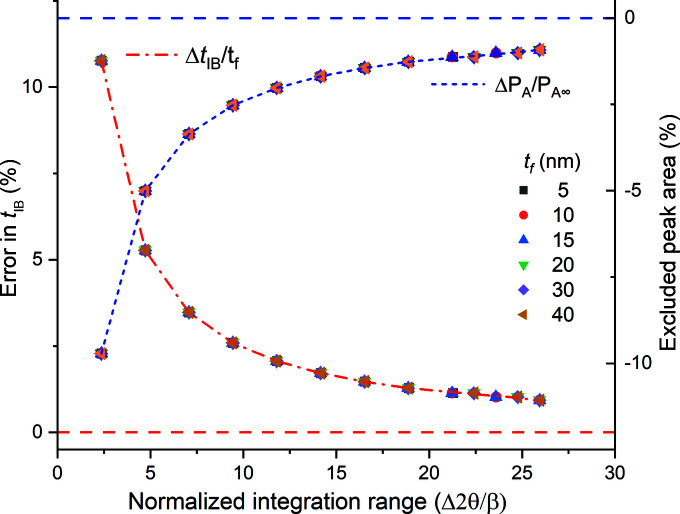
The variation in the fractional errors in *t*_IB_, and the corresponding excluded peak areas with normalized integration range for various film thicknesses *t*_f_.

To check the generality of these observations, we computed the Patterson profiles for the 111 peaks of single-crystal thin films of various thicknesses and repeated the computations described above for each profile. The results are summarized in Fig. 5 ▸, where the fractional thickness error Δ*t* = (*t*_f,IB_ − *t*_f_)/*t*_f_ and the corresponding Δ*P*_A_ values [equation (9)] are plotted versus the normalized integration range for all cases. Both of these parameters are independent of size. In all cases the fractional error obtained from using the integral breadth obtained by fitting a single Gaussian to the central peak yielded an identical fractional error corresponding to the first Δ*t*_f,IB_ datum (integration of the central peak). For all films, when *t*_f,IB_ was computed using the definite integral 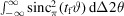 , the result had zero fractional error.

We conclude that it is challenging to obtain the true integral breadth from fitting a single function of any approximate form to the central peak of a radial scan. Numerical integration of an experimental profile over the largest possible angular range might yield better results. However, given the asymptotic approach of the integrated intensities to the definite integral values in Fig. 5 ▸, errors of several percent would be expected in the computed thickness values, since the presence of neighbouring peaks or experimental issues might limit the angular scan range.

### 2.2. Diffraction from single-crystal cylinders and spheres

In the case of a cylinder of height *L*_cy_ and base radius *R*_cy_, where 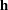 is along the radial direction, equation (4) becomes

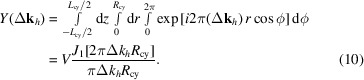

The corresponding diffracted intensity in terms of deviation from the Bragg angle Δ2θ is

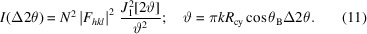

The half-maximum intensity is reached when 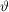 = ±0.808. The FWHM (β) value can be expressed in terms of the base diameter *D*_cy_ of the cylinder as

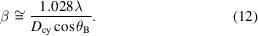

Likewise, for diffraction from a single-crystal sphere of radius *R*_sp_ (diameter *D*_sp_), we obtain

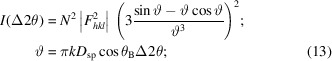




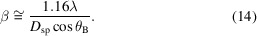




**Figure 6:**
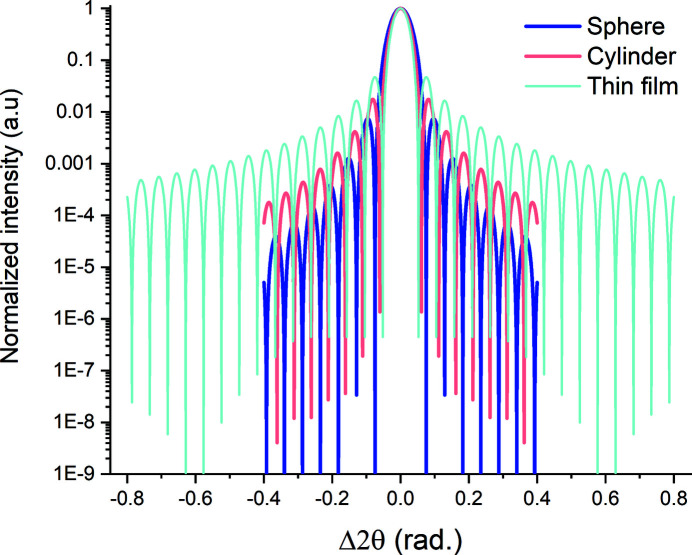
Computed radial scan profiles of the 220 reflections for single-crystal film, cylinder and sphere geometries (*t*_f_ = *D*_cy_ = *D*_sp_ = 5 nm). λ = 2.2909 Å in all cases.

Equations (11) and (13) yield radial scan profiles similar to the thin-film profiles [equation (6)]. In Fig. 6 ▸ we plot the 220 radial scans computed with λ = 2.2909 Å for a cylinder and a sphere, with *D*_cy_ = *D*_sp_ = 5 nm, and for a thin film, with *t*_f_ = 5 nm. All three profiles have a central (primary) peak of unit amplitude bracketed with symmetric thickness fringes. The FWHMs of the central peaks are slightly different for the three sample geometries, commensurate with the differences in the Scherrer coefficients in equations (7*b*)[Disp-formula fd7b], (12) and (14). The rates of decay of the thickness fringes are significantly different, with those belonging to the sphere profile extinguishing fastest. The periods of the zeroes of the thickness fringes are equal for all three cases, *i.e.* the thickness-fringe zeroes are independent of shape. Consequently, equation (7*a*)[Disp-formula fd7a] can be used directly for computing *D*_cy_ and *D*_sp_.

In contrast to the thin-film case [equation (6)], the integral breadths of the peaks described by equations (11) and (13) cannot be used directly in equation (7*c*)[Disp-formula fd7c] to calculate *D*_cy_ and *D*_sp_ since these equations are not normalized (while the intensities generated by both functions are in the range between 0 and 1, their norms are not unity). In the case of a cylinder, the integrated intensity of the rocking curve is given by

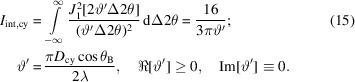




Thus, the integral breadth obtained from the rocking curve of the cylindrical sample is related to the base diameter of the cylinder by

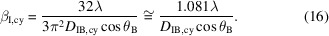




For the case of a spherical sample, a similar treatment yields

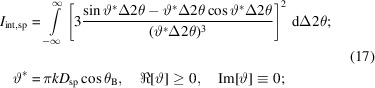




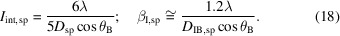




Consequently, when the Patterson equations are used to simulate radial scan profiles from spherical or cylindrical single-crystal samples, a ‘Scherrer-like’ proportionality constant (shape constant) must be included when linking the integral breadth of these profiles to the diameters of such samples, *D*_IB,cy_ and *D*_IB,sp_, even when the radial scans are integrated over infinite angular ranges, 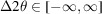 .

**Figure 7:**
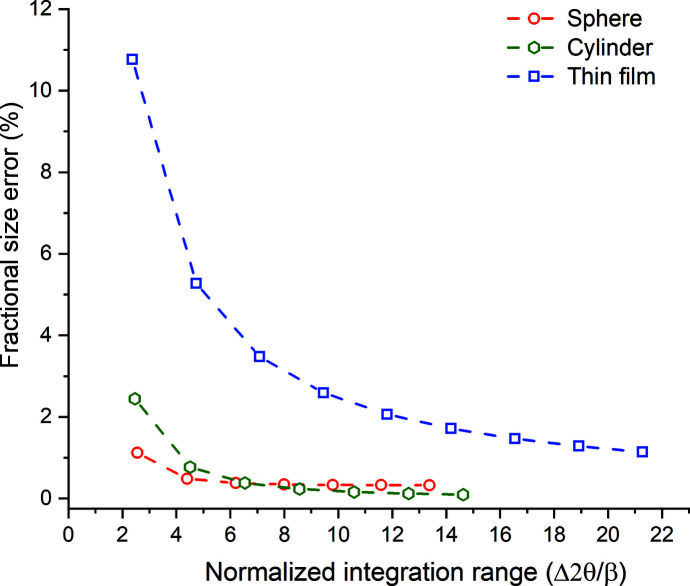
The fractional error in the crystal size (film thickness, cylinder diameter, sphere diameter) computed from the integral breadth of the corresponding radial scan as a function of the normalized integration range.

Integration over a narrower angular range^5^ will introduce a truncation error in the diameters *D*_IB_ even when such a proportionality constant is used (Table 2 ▸). For a given normalized integration range this error will be smaller for cylindrical and spherical single crystals than single-crystal thin films, since the central peak of these patterns contains a larger fraction of the diffracted intensity (Fig. 7 ▸). For both of these geometries, evaluating the diameter from a Gaussian fit with the theoretical Scherrer constants to the central peak only yielded ∼2% error (Table 2 ▸). This small error is due to the Gaussian peak approximation. If we calculate the Scherrer coefficients by correlating the FWHMs of the simulated peaks fitted with Gaussians to the actual sphere diameters used in the simulations, we obtain *C* = 1.12 and 0.99 for the sphere and cylinder coefficients, respectively. The corresponding theoretical values for these geometries obtained from the exact functional forms are *C* = 1.16 and 1.03 (Patterson, 1939*a* ▸,*b* ▸).

## 3. Fourier analysis

Fourier decomposition is also widely used to obtain crystallite size, lattice strain and stacking fault distributions from diffraction peak profiles. The fundamental theory and its extensions are widely discussed in textbooks (Taylor, 1961 ▸; Warren, 1969 ▸; Klug & Alexander, 1974 ▸; Schwartz & Cohen, 1987 ▸; Guinier, 1994 ▸) and in journal articles (Warren & Averbach, 1950 ▸, 1952 ▸; Langford *et al.*, 2000 ▸; Ida *et al.*, 2003 ▸; Lucks *et al.*, 2004 ▸; Scardi & Leoni, 2006 ▸; Mittemeijer & Welzel, 2008 ▸; Cline *et al.*, 2020 ▸). The basic approach consists of correcting the measured diffraction profiles of one or more diffraction peaks for background, peak overlap and instrumental effects. These corrected profiles are then decomposed into their Fourier components, from which the relevant parameters are computed. In the absence of strain (or other sample-related) broadening, the Fourier cosine coefficients of any reflection can be used to determine either the distribution of crystallite sizes or an average crystallite size for a polycrystalline powder. For a powder sample with unimodal size and shape distribution, or for a kinematically scattering single crystal, the average crystallite size determined from Fourier decomposition is the average chord size along the diffraction vector 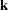 , which depends on the real-space shape of the crystallite, the diffraction geometry and the Miller indices of the reflection used in the analysis (Guinier, 1994 ▸). In what follows we apply the basic Fourier analysis directly to synthetic peak profiles generated using the Patterson equations for thin-film, spherical and cylindrical samples. Rather than reprising the theory, which is readily accessible in the literature, we will provide a comprehensive discussion of the actual steps of the basic analysis which yields the average dimension along 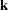 , with further details about the use of the Fourier transform provided in Appendix *A* [App appa].

The discrete peak profiles used in the analysis were computed using a simple *Mathematica* notebook (Wolfram, 2021 ▸), which output the simulated diffraction peak intensities as [*I_j_*, Δ2θ_*j*_] data sets with *M* equidistant δ2θ points. Here, *I_j_* is the *j*th intensity point at angular position Δ2θ_*j*_ = j*δ2θ, where 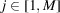 . We then used a discrete Fourier transform (DFT) formulation *restricted to real numbers*, 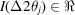 , to express each peak as a periodic function *I*_F_(Δ2θ) in the angular range (0, *M*δ2θ),

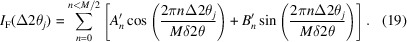

Here, 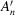 and 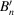 are, respectively, the Fourier cosine and sine coefficients of order 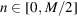 , and *I*_F_(Δ2θ_*j*_) is the (Fourier) synthesized intensity at angular position Δ2θ_*j*_. While the profiles generated by the Patterson equations are centred at Δ2θ = 0, the peak profiles obtained from equation (19) are shifted by half of the angular range and have peak maxima at Δ2θ = (*M*δ2θ)/2. This shift does not affect the analysis results.

**Figure 8:**
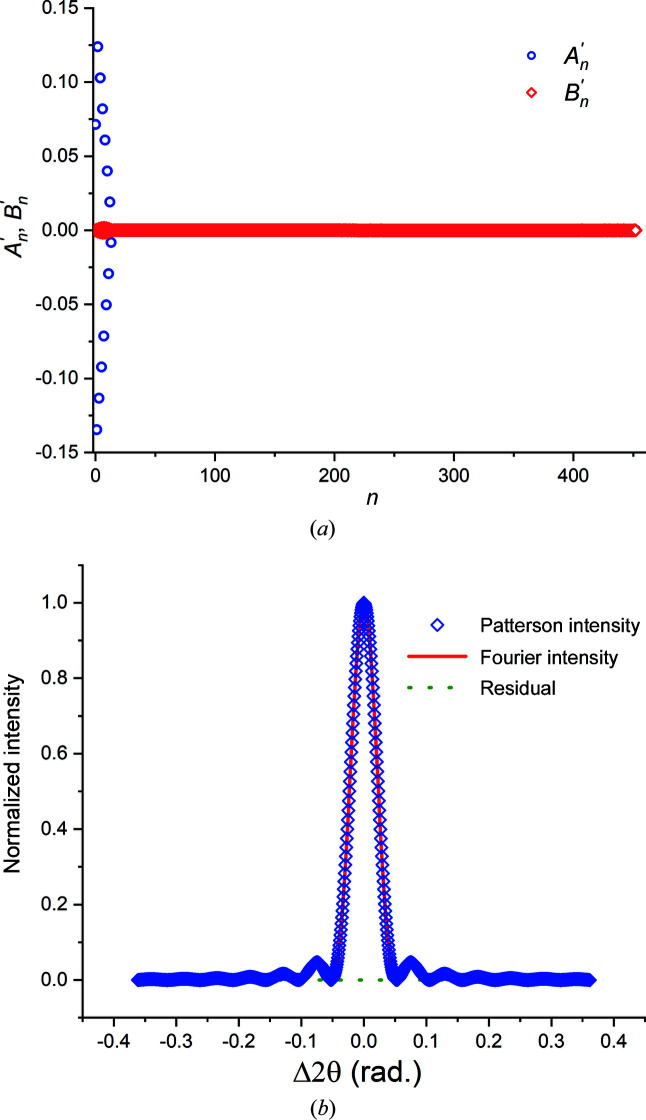
(*a*) The cosine and sine coefficients 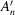 and 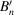 computed for the 111 reflection of a hypothetical 5 nm thick Au thin film. (*b*) The intensity distribution *I*_F_(Δ2θ), computed using the coefficients shown in panel (*a*), superimposed on the original (simulated) data set.

In Fig. 8 ▸(*a*), the cosine and sine coefficients 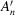 and 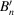 computed for the 111 reflection of a hypothetical 5 nm thick Au thin film using Cr *K*α radiation are shown for *n* ≤ 452. The 111 peak profile was sampled at 905 steps (*M* = 905), 131 of which were in the central peak. For all *n*, 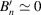 , reflecting the symmetry of the Bragg peak. The function *I*_F_(Δ2θ) computed using these coefficients showed good fidelity to the actual [*I*, δ2θ] data set [Fig. 8 ▸(*b*)]. The maximum residual normalized intensity difference between the actual and re-calculated intensity data sets was 5 × 10^−7^.

**Figure 9:**
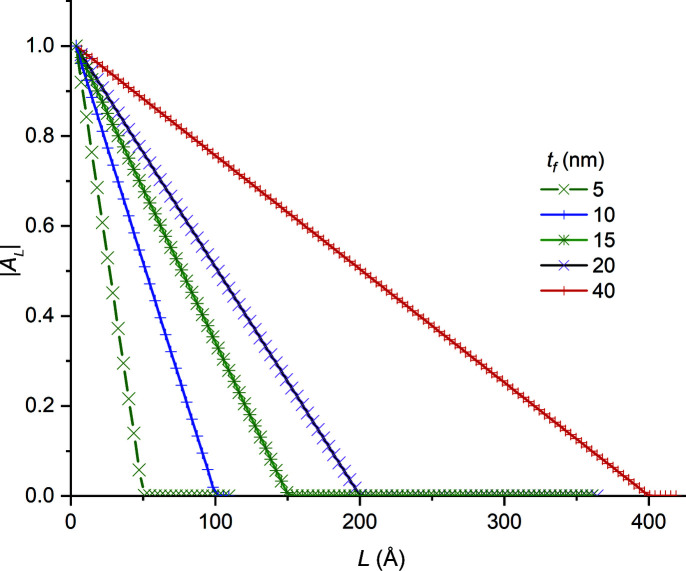
The normalized Fourier cosine coefficients, |*A_L_* |, of Au thin films of various thicknesses, computed from their 111 reflections and plotted versus column length *L*. The intercepts with the abscissa yield the film thicknesses.

To obtain the thin-film thickness, we computed the column length *L* (the length of the columns of the unit cells in the direction of the diffraction vector; Guinier, 1994 ▸) as 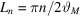 for each Fourier order *n*, and the corresponding absolute values of the normalized cosine coefficients,^6^ 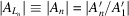 . Here, 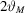 is the full range for 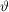 [equation (6)] over which the Patterson intensities were computed. In what follows we will omit the order subscript on the column length for brevity, following the common usage in the literature. Fig. 9 ▸ depicts the variation in |*A_L_* | versus *L* for the full set of thin-film simulations. We observe that

(i) for all film thicknesses, |*A_L_* | versus *L* varies linearly for *L* < *t*_f_, and

(ii) least-squares lines fitted to |*A_L_* | versus *L* data over the entire 0 < *L* < *t*_f_ range, or any of its subsets, intersect the abscissa at *L* = *t*_f_.

These observations agree with our experimental results obtained from single-crystal silicon-on-insulator thin films (Ying *et al.*, 2009 ▸).

**Figure 10:**
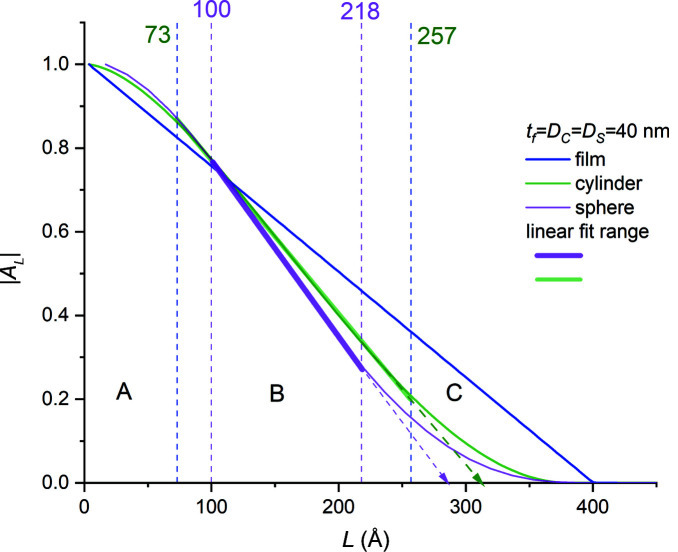
|*A_L_* | versus *L* plots for the 220 reflections of the single-crystal samples shown in Fig. 6 ▸. The intercepts of the linear region with the abscissa (dashed arrows) yield the volume-averaged chord length along the scattering vector **k**. The outer and inner dashed line pairs show the boundaries of the linear fit regions for the cylindrical and spherical samples, respectively. The *A_L_* terms for *L* = 0 are not included in the plot due to the DFT formulation used [Appendix *A* [App appa], equation (24)].

In contrast to the thin-film plots shown in Fig. 9 ▸, |*A_L_* | versus *L* plots for spheres and cylinders exhibit sigmoid-like profiles (Fig. 10 ▸) with quasi-linear central regions. For a cylinder simulation with *D*_cy_ = 40 nm, the linear |*A_L_* | versus *L* range is approximately 46% of the linear range for the 40 nm film. For a sphere simulation with *D*_sp_ = 40 nm, the linear range drops to 30%. Thus, the geometry of the scattering volume (or, more precisely, the form of the distribution of chord lengths along the diffraction vector within the crystallite volume) introduces nonlinearities into the initial and final ranges of the |*A_L_* | versus *L* plots. Consequently, the curvature in the initial part of such plots cannot be ascribed solely to the traditional ‘hook effect’ (Warren, 1969 ▸) caused by background subtraction issues or incomplete measurement ranges.

The presence of these nonlinear regions requires the use of a consistent technique for defining the linear least-squares fit range to ensure precise determination of the desired crystal dimension. For this purpose we computed the (local) slope of the line segments for each adjacent pair of normalized cosine coefficients (|*A_L_* |_*j*_, |*A_L_* |_*j*−1_), and in the regression analysis we included only those pairs yielding local slopes within 10% of the mean slope of the line fitted to the middle ‘linear’ region. These regions are highlighted in Fig. 10 ▸. The lines fitted to these regions intersected the abscissa at *L* values quite close to the average chord lengths of the cylinder and sphere shapes along the diffraction vector (Guinier, 1994 ▸),

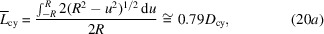




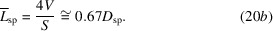

For symmetric radial scans obtained from single-crystal thin films, the average cord length is identically equal to the film thickness,

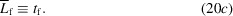




**Figure 11:**
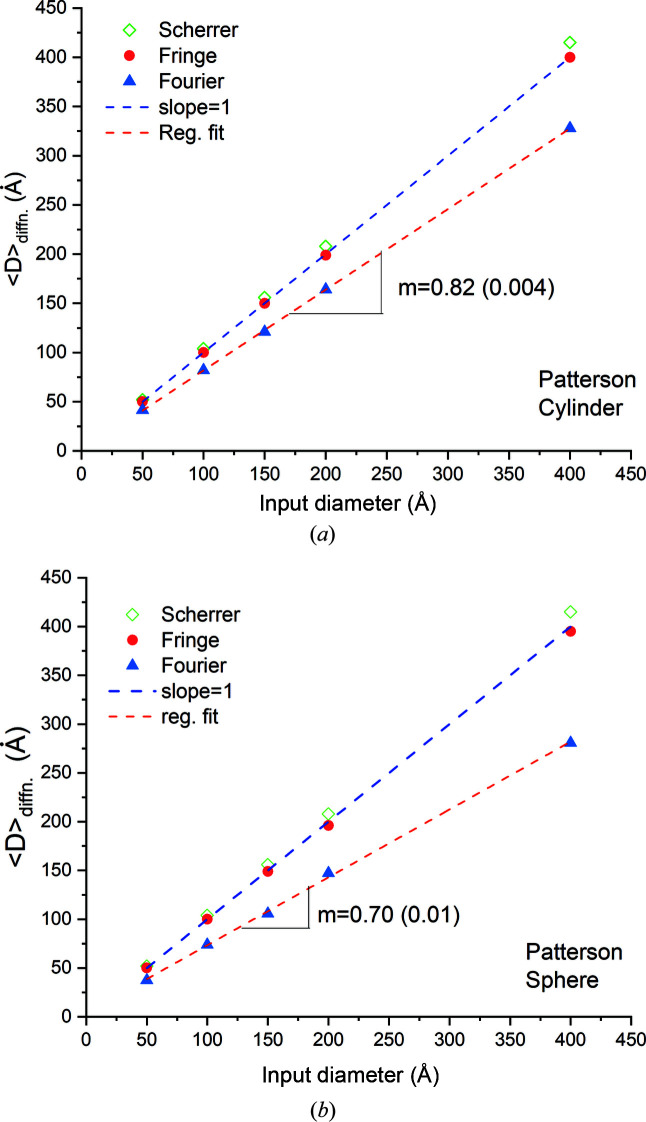
Plots showing the variation in the (average) dimension along the scattering vector of (*a*) single-crystal cylinders and (*b*) single-crystal spheres obtained from diffraction analysis, plotted versus input diameter. The Scherrer and thickness-fringe analyses yield the maximum real-space dimension. Fourier analysis yields the volume-averaged chord length.

To obtain better statistics we applied the Fourier formalism to spheres and cylinders with diameters ranging from 5 to 40 nm and obtained the Fourier-averaged cylinder and sphere diameters, 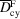 and 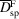 , respectively, from the intercepts of the linear regression lines. As shown in Figs. 11 ▸(*a*) and 11 ▸(*b*), both 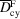 and 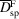 depend linearly on the respective (geometric) diameters, with 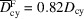 and 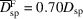 . In both cases there is a ∼4% deviation from the theoretical values predicted by equations (20*a*)[Disp-formula fd20a] and (20*b*)[Disp-formula fd20b] for the ranges analysed. This error is larger than the ‘fit’ errors assigned to the slopes by regression analysis.

We were not able to obtain satisfactory diameter values when we used the initial regions of the |*A_L_* | versus *L* plots for these geometries as suggested in the literature. We note that selection or exclusion of a few points at the start and/or end of the linear range could change the slope by an additional 5% or more, with concomitant changes in the 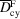 and 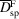 values. Consequently, for these specimen geometries, even if all other broadening sources were successfully eliminated and there was no surface roughness, experimentally determined particle sizes could have uncertainties in the region of ±5%.

### 3.1. Multiple peak comparison

**Figure 12:**
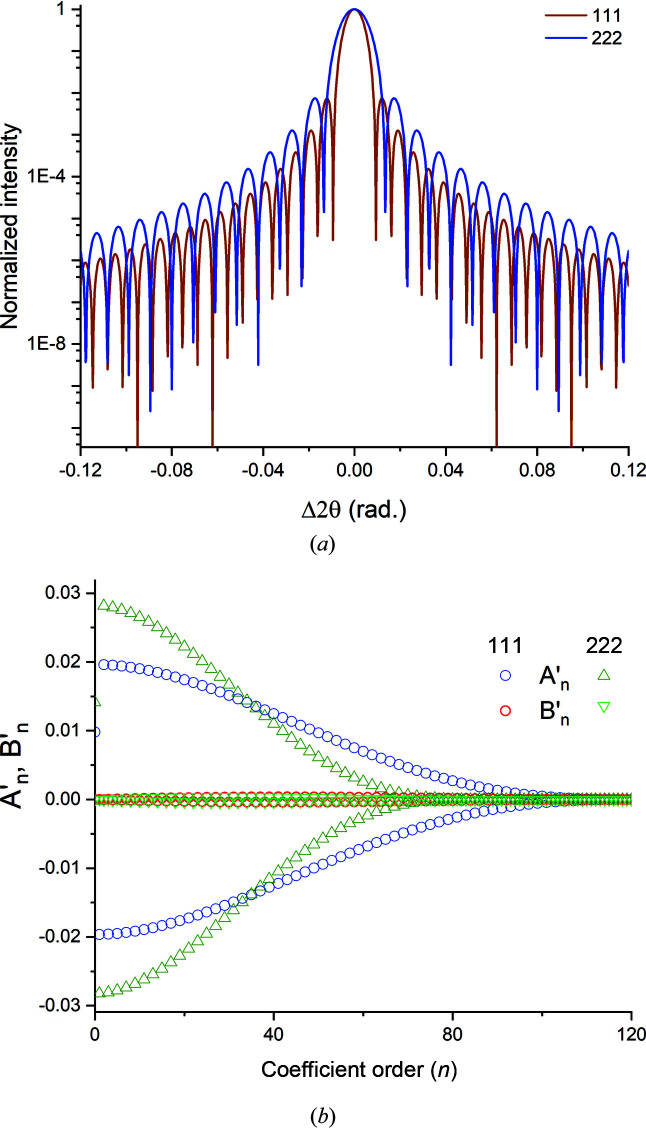
(*a*) Computed 111 and 222 radial scans of a hypothetical single-crystal Au sphere 40 nm in diameter (λ = 2.2909 Å). (*b*) The cosine and sine coefficients, 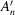 and 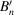 , computed for the 111 and 222 reflections depicted in panel (*a*).

As a final test of the Fourier analysis formalism we investigated the effect of the curved |*A_L_* | versus *L* regions in two orders of the same reflection, postulating that, if our approach to determining the ‘linear region’ is correct, both reflections should yield the same average chord length within the ‘fit’ error since there is no strain broadening. Fig. 12 ▸(*a*) shows the 111 and 222 reflections of a 40 nm Au single-crystal sphere computed using equation (13) with λ = 2.2909 Å. Due to the cosθ_B_ term, the FWHMs of the primary peaks and the period *T* of the fringe minima are different. These differences are reflected in the (raw) cosine and sine coefficients, 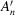 and 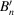 , of the Fourier series corresponding to these peaks [Fig. 12 ▸(*b*)].

**Figure 13:**
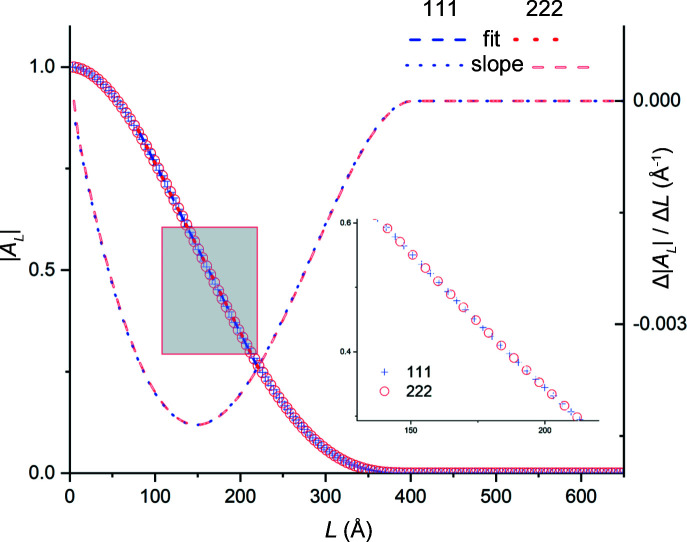
Normalized Fourier cosine coefficients, |*A_L_* |, for the 111 and 222 reflections shown in Fig. 12(*b*) plotted versus column length *L*. The right ordinate corresponds to the local slope values. The inset shows an expanded view of |*A_L_* | in the linear region (shaded box).

In contrast to Fig. 12 ▸(*b*), the normalized cosine parameters |*A_L_* | for the two reflections are very close in value and exhibit similar *L* dependencies (Fig. 13 ▸). Also, the local slopes of the two curves are almost identical. Consequently, the effective sphere diameters [equal to the mean chord length of the sphere, equation (20*b*)[Disp-formula fd20b]] obtained from extrapolation of the linear portions of these *A_n_* versus *L* plots are very close for the two reflections (Table 3 ▸), reflecting the absence of (order-dependent) strain broadening in our model.

## 4. Summary

In this study we first simulated stand-alone diffraction peaks from kinematically scattering strain-free single crystals in the shape of thin films, cylinders and spheres using their Patterson equations. These simulated peak profiles were centred at the exact Bragg angle, with tails extending to 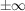 , and were free from other sample-based broadening effects such as dis­locations, stacking faults, strain, instrumental broadening or background profiles. We then computed the relevant crystal dimension, thickness or diameter, along the scattering vector 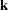 for these crystals using (i) the FWHM (β) and integral-breadth forms of the Scherrer equation, (ii) the period of the thickness fringes, and (iii) single-peak Fourier analysis. The difference between the dimension input into the simulation and that obtained from a given technique was taken as a measure of the minimum uncertainty (error) associated with the results from the particular technique. To obtain better statistics this analysis was conducted for a range of crystal sizes. We observed the following:

(i) The best accuracy was achieved when the zeroes of the thickness fringes were used to compute the crystal dimension along 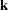 . For well defined fringes, sub-ångström precision in the relevant dimension could be achieved. This observation agrees with our previous experimental work on silicon-on-insulator single-crystal thin films.

(ii) The use of a Gaussian function to approximate the central peak of the diffraction profile introduced errors into the crystal dimensions obtained from both forms, FWHM and IB, of the Scherrer equation.

(*a*) Use of the Gaussian FWHM in the Scherrer equation yielded approximately 5% error in the computed crystal size. This error was independent of shape.

(*b*) A shape constant, not equal to one, was needed in the computation of the diameters of cylindrical and spherical crystals using integral-breadth values, since the Patterson equations describing the peak profiles for these shapes are not normalized functions, even though the computed intensities range between zero and unity.

(*c*) Even when such a shape constant was used, if the relevant crystal size was computed from the integral breadth of a Gaussian function fitted to the central peak, errors in the range of 10% (for thin films) to 2% (for spheres) were observed. These errors are due to the approximation of the diffraction line shape with the Gaussian function and depend on the shape of the crystal. Similar errors are possible when other approximation functions (such as pseudo-Voigt, Pearson VII or Lorentzian formulations) are employed.

(iii) Single-peak Fourier techniques also yielded shape-dependent crystal size errors.

(*a*) For perfect thin films, the variation in |*A_L_* | versus *L* was linear for all thin-film dimensions used in the study (Fig. 9 ▸). Average thickness values computed from the intercepts of these plots on the abscissa had negligible errors.

(*b*) |*A_L_* | versus *L* plots computed for cylindrical and spherical specimens had sigmoid-like |*A_L_* | versus *L* profiles (Fig. 10 ▸).

(1) The initial concave-down curvature in these plots was caused by the shape of the crystal. It is not due to the traditional ‘hook’ effect and could not be corrected using traditional approaches. The Fourier transform of a diffraction peak from a crystallite yields the auto-correlation function of the crystallite along the scattering vector. This is analogous to the distribution of signal energy across frequencies in signal processing and is proportional to the average number of unit cells across the crystallite cross section participating in diffraction for the particular scattering angle (expressed as the deviation from the exact Bragg angle Δ2θ). This number is constant when the crystal is a thin film. For spherical or cylindrical samples, the variation in this number with Δ2θ is never truly linear. Thus, the shape of the |*A_L_* | versus *L* plots will depend on the shape of the crystal; if the shape of the crystal is non-symmetric along the scattering vector with Δ2θ, the |*A_L_* | versus *L* plot will also be non-symmetric.

(2) The presence of these nonlinear regions necessitated fitting the central region of these plots with a line to obtain the relevant crystal diameters. Since the definition of these linear regions was somewhat arbitrary, selection or omission of a few points could contribute errors of about 5% in the extrapolated average crystal diameter.

(iv) As shown in Appendix *A* [App appa], when Fourier-decomposition programs which are optimized to treat real number data are applied to a peak profile with *M* intensity points, the cosine and sine coefficients for 1 ≤ *n* < *M*/2 will be twice the magnitude of the coefficients obtained from standard programs, while the coefficients for the zeroth order and the Nyquist frequency (*n* = 0, *n* = *M*/2) will be identical. For such codes the zeroth-order (raw) cosine coefficient 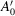 must be corrected if it is to be included in the analysis.

(v) For Fourier analysis, all other considerations being equal, it was much better to use a broad angular range with fewer steps, rather than a narrow angular range with finer steps. If a narrow range is used, using ‘padding’ might enable the recovery of useful data from the analysis (Appendix *A* [App appa]).

## 5. Conclusions

Even for the best case, where all confounding issues have been eliminated, classical diffraction-based particle size values, with the exception of those from thickness zeroes, have fractional errors of around 5% for the coherent domain size along the scattering direction. This value is a lower bound; the presence of other line-broadening sources, instrumental effects or emergent scattering artefacts such as the nanoparticle size error (Xiong *et al.*, 2018 ▸, 2019 ▸; Kaszkur, 2019 ▸) will cause larger uncertainties. On the basis of our results we recommend that, once a diffraction pattern has been corrected for instrumental broadening, all domain size formalisms, *i.e.* thickness fringe zeroes,^7^ FWHM and IB formulations of the Scherrer equation, and Fourier decomposition, should be applied to all available reflections; these results should then be evaluated together. If all values agree within experimental error, then the sample probably exhibits a uniform chord length distribution along the scattering vectors. If there is significant divergence among these results, the ‘average particle size(s)’ obtained might not be reliable. In such cases, if other sources of broadening can be eliminated, more sophisticated approaches involving domain size distributions such as those described by Scardi & Leoni (2006 ▸) might be undertaken. 

## Figures and Tables

**Figure 14:**
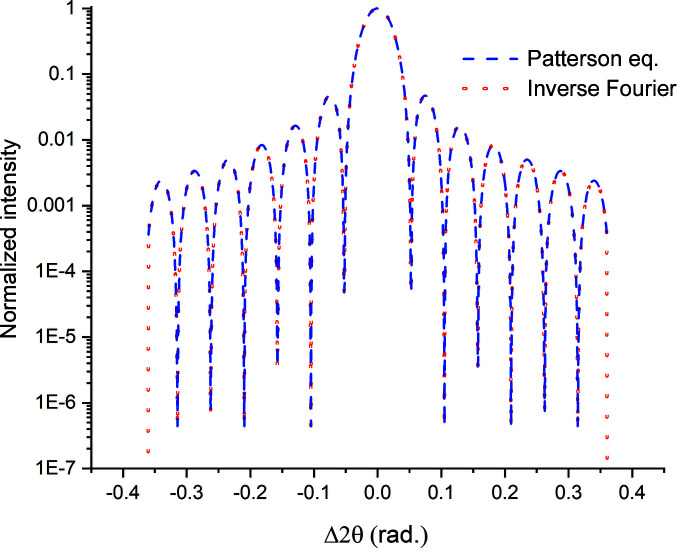
The 111 peak profile computed from the Patterson equation for a hypothetical 50 Å thick Au thin film using Cr *K*α radiation. There are *M* = 905 points in this plot, and the profile contains 98.6% of the integrated intensity of the theoretical peak. The red dotted line depicts the profile obtained from the inverse Fourier transform.

**Figure 15:**
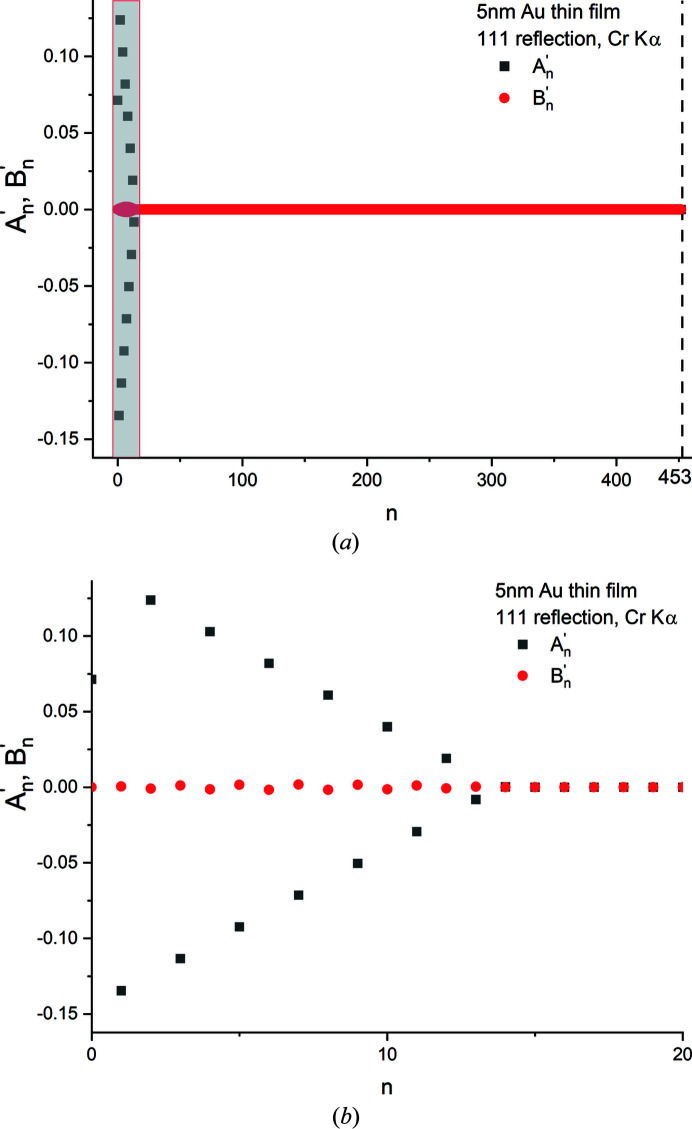
(*a*) The raw Fourier coefficients obtained from the decomposition of the profile in Fig. 14 using an algorithm optimized for real numbers only. (*b*) The raw Fourier coefficients in the shaded area of panel (*a*). The zeroth-order cosine coefficient is much lower in magnitude than would be expected solely from the missing peak area.

**Figure 16:**
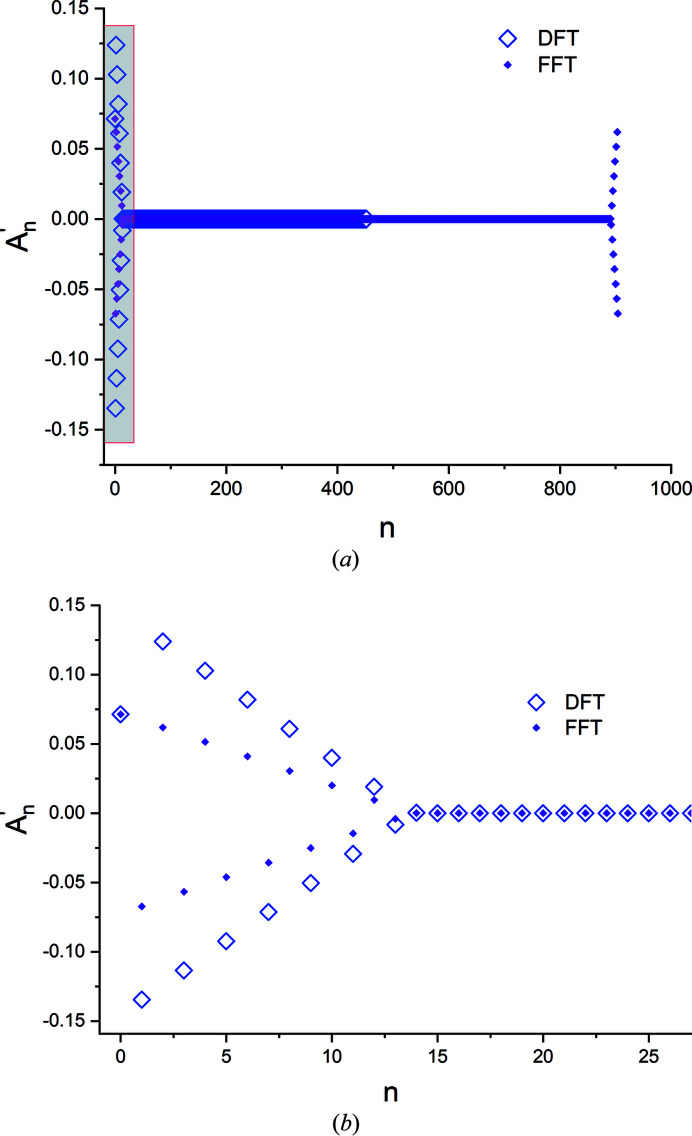
(*a*) The raw Fourier cosine coefficients obtained from the decomposition of the profile in Fig. 14 using a general FFT algorithm (solid dots). All coefficients obtained from the DFT code (open diamonds) are overlaid for comparison. (*b*) An expanded plot of the shaded box in panel (*a*). In this range, with the exception of the zeroth-order coefficient 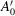 , the 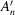 from the DFT code are twice those obtained from the FFT code.

**Figure 17:**
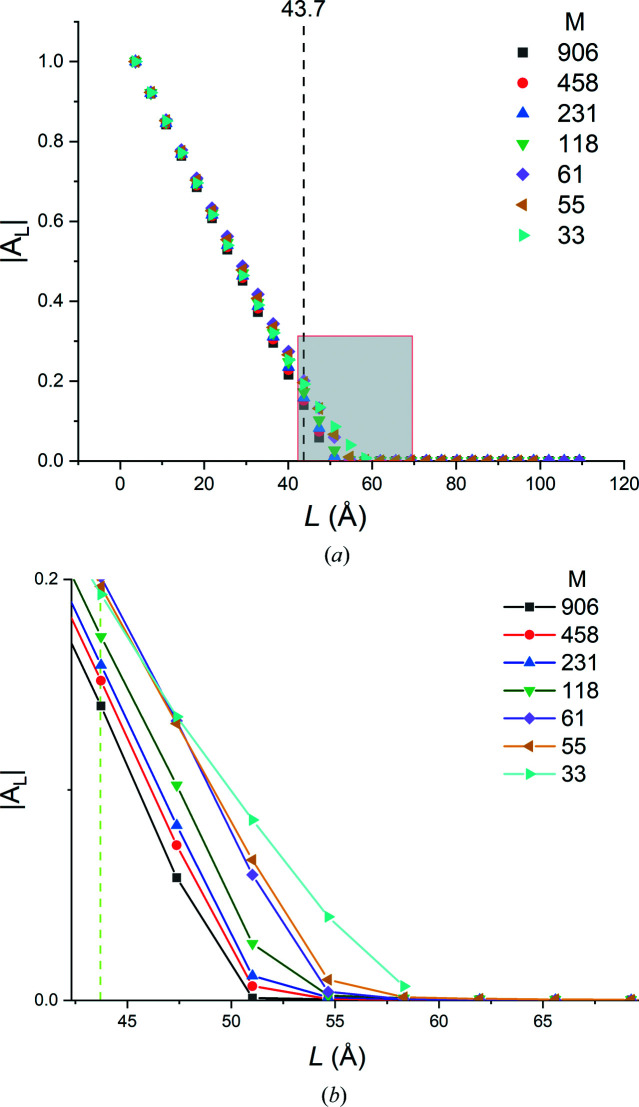
(*a*) |*A_L_* | versus *L* plots for the simulations described in Table 4 ▸. (*b*) An enlargement of the shaded area in panel (*a*).

**Figure 18:**
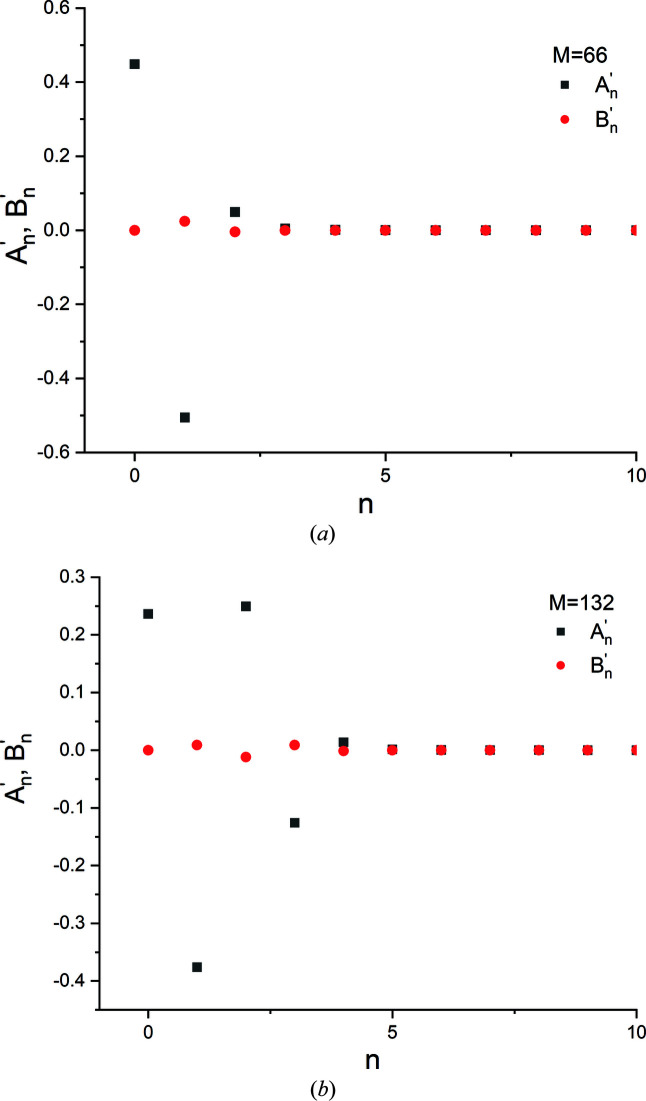
The Fourier coefficients 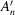 and 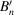 for the Bragg profiles described in rows 2 and 3 of Table 5. These profiles contain (*a*) the central peak only with 66 intensity data points and (*b*) the central peak plus a pair of thickness fringes with 132 discrete data points, respectively. For the first case the number of useful cosine coefficients is inadequate for further analysis.

**Figure 19:**
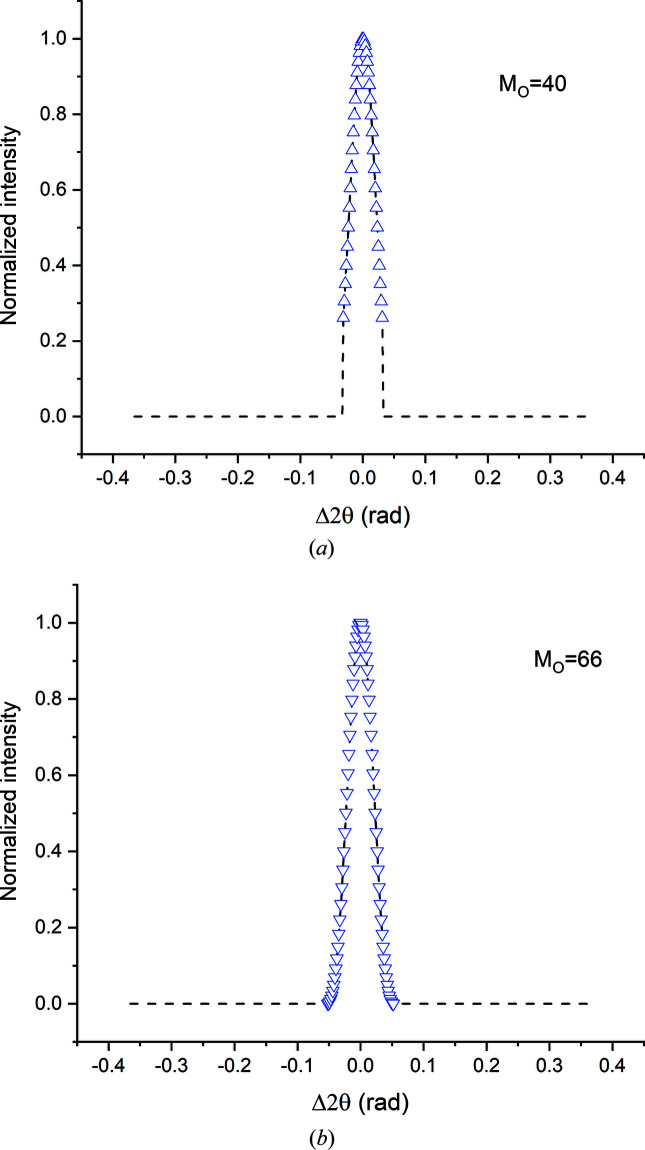
‘Padded’ profiles containing (*a*) the top 75% and (*b*) 100% of the primary peak, with 40 and 66 original intensity values (*M*_o_ = 40 and 60). The actual data points are shown as triangles. The dashed lines indicate the ranges padded with zero intensity values.

**Figure 20:**
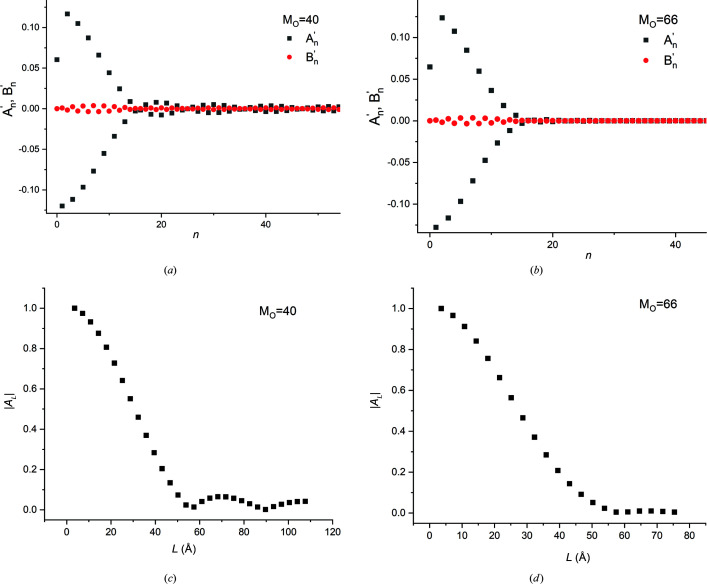
(*a*), (*b*) The variation in the (raw) Fourier coefficients 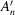 and 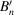 with coefficient order *n*, and (*c*), (*d*) the variation in the normalized cosine coefficient |*A_L_* | with column length *L*, for the padded profiles containing (*a*) the top 75% and (*b*) 100% of the primary peak, with 40 and 66 original intensity values, respectively.

**Table 1:** Film thickness values computed from the integral breadth *t*_I_ for the profile shown in Fig. 2 ▸ (*t*_f_ = 50 Å, λ = 2.2909 Å) using either integration or Gaussian fits over various angular ranges The thickness values *t*_β_ obtained from the Scherrer equation using the FWHM of the central peak for the various fits are also shown.

		No. of fringe peaks each side			
	Central peak	One	Two	Three	Infinity
*t*_f,IB_ (Å), integration	55.4	52.6	51.7	51.3	50.00
*t*_f,IB_ (Å), Gaussian fit	55.4	53.0	52.2	51.5	N/A
*t*_f,β_ (Å), Gaussian fit	52.4	52.3	52.3	52.2	N/A
					
*t*_f,*z*_ (Å), fringe zero period		50.0			

**Table 2:** Cylinder and sphere diameters obtained from the FWHM (*C* = 1.028 and 1.16, respectively) and integral breadths of Gaussians fitted to the central peaks of the Patterson profiles

		*D* model (nm)					
		5	10	15	20	30	40
Cylinder	*D*_cy_ FWHM	5.2	10.4	15.6	20.8	31.1	41.5
	*D*_cy_ IB	5.1	10.3	15.4	20.5	31.2	41.0
Sphere	*D*_sp_ FWHM	5.1	10.3	15.4	20.6	30.9	41.2
	*D*_sp_ IB	5.0	10.1	15.1	20.2	30.6	40.4

**Table 3:** Effective diameters (standard deviations) computed from simulated 111 and 222 radial scans from a 40 nm diameter Au single-crystal sphere using the FWHM (*D*_β,sp_), thickness fringe period (*D*_*z*,sp_) and Fourier single-peak analysis, 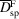

Reflection	*D*_β,sp_ (Å)	*D*_*z*,sp_ (Å)	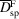 (Å)
111	413 (1)	399 (1)	283 (1)
222	413 (1)	399 (1)	283 (1)

**Table 4:** The effect of the step size at fixed angular range (full range 0.73 rad = 16.5β) on the film thickness values obtained from Fourier analysis of the 111 Bragg profiles of a 50 Å thick Au film sampled with 906 to 33 steps

δ2θ [rad, (°)]	δ2θ/β	*M*	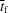 (Å)
0.0008 (0.046)	0.028	906	50.2
0.0016 (0.092)	0.036	458	50.9
0.0032 (0.183)	0.072	231	51.3
0.0064 (0.367)	0.144	118	52.0
0.0128 (0.733)	0.289	61	53.8
0.0144 (0.825)	0.325	55	53.2
0.0256 (1.467)	0.577	33	52.3

**Table 5:** The effect of the angular frame range at fixed step size (δ2θ = 0.90° = 0.00160 rad) on the film thickness values obtained from Fourier analysis of the 111 Bragg profiles of a 50 Å thick Au film The frame ranges studied ranged from 1.4 to 16.5β.

Frame range (× β)	*M*	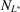 (*n* ≥ 1)	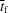 (Å)	Notes
1.4	40	1	N.A.	Top 75% of central peak (CP)
2.4	66	1	N.A.	CP
4.7	132	3	50.1	CP + pair of fringe peaks (FP)
7.1	198	6	50.6	CP+ two pairs of FP
9.5	264	8	50.4	CP + three pairs of FP
16.5	458	14	50.9	Full range (Fig. 14)

**Table 6:** The effect of padding on the film thickness values obtained from Fourier analysis of the 111 Bragg profiles of a 50 Å thick Au film

*M*_o_	Real frame range (× β)	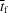 (Å)
40	1.4	51.7
66	2.4	47.1
132	4.7	49.7
198	7.1	49.9

## Footnotes

1 Some publications (Matyi *et al.*, 1987 ▸; Leoni, 2019 ▸) identify this dimension as a ‘reciprocal-space length’. This issue is discussed later in the article.

2 In what follows we neglect the multiplier terms  in the Patterson intensity equations for brevity. Consequently, the unit cell of the particular crystal will be represented in the peak shape only by the Bragg angle θ_B_ of the particular reflection being simulated.

3 In equations (7*a*)[Disp-formula fd7a]–(7*c*)[Disp-formula fd7b] [Disp-formula fd7c] we differentiate between the thicknesses obtained by these techniques using subscripts. In the absence of errors all of these terms must be equal to the real-space film thickness: *t*_f,*z*_ = *t*_f,β_ = *t*_f,IB_ ≡ *t*_f_.

4 The Patterson formulations are based on ideal geometric particle shapes with smooth surfaces. The presence of surface roughness will exacerbate such errors and increase the uncertainty associated with the trueness of such values.

5 Since equation (17) is not well behaved in the vicinity of Δ2θ = 0, numerical integration of the function in this range requires care.

6 In general, *A_n_* (the normalized cosine coefficient of order *n*) is not equal to the (raw) cosine coefficient  . In our calculations we used  for normalization (instead of the zeroth-order coefficient  normally employed for this purpose in the literature) due to the form of equation (19). Further details are supplied in the Appendix[App appa].

7 This would be possible for single-crystal samples or polycrystalline samples with large coherently scattering domains (Saenger & Noyan, 2001 ▸).

## Appendix A Fourier decomposition of Bragg peaks

This analysis starts with the measurement (or computation) of a given peak profile spanning an angular range Δ2θ_FR_. In most literature treating Fourier analysis of signals, this is termed a ‘frame’. We assume that the scattered intensities in this frame are sampled at *M* equidistant angular positions, yielding an array of intensity versus angular positions, [*I_j_*, Δ2θ_*j*_], where Δ2θ_*j*_ = *j*δ2θ,  , and Δ2θ_FR_ = *M*δ2θ. This intensity profile is corrected for instrumental effects and background as needed, and then the intensity values are normalized such that  . The first goal of the analysis is the determination of a Fourier interpolation function *I*_F_(Δ2θ), which matches the (normalized) intensities for all Δ2θ_*j*_,

where *n* is the order of the Fourier coefficients. Since at least two data points are required per period to resolve a wave, the highest unique ‘frequency’ component resolvable in the Fourier transform of the diffraction pattern is at *n*_NL_ = *M*/2, which is termed the Nyquist limit (Smith, 1999 ▸; Avillez *et al.*, 2018 ▸).^8^

The cosine and sine Fourier coefficients are related to the complex coefficients through

The complex Fourier coefficients can be computed from

The corresponding cosine and sine coefficients can then be computed from equation (22). In the literature, equations (21) and (23) are termed the discrete Fourier transform (DFT) and the inverse discrete Fourier transform, respectively.

### A1. Decomposition algorithms

In practice, freely available or commercial programs are used to compute cosine and sine coefficients. Most of these programs have several options for data conditioning and use different algorithms. Consequently, the number and magnitude of coefficients obtained from different programs may not agree. We compared three programs for this purpose.

In the first case, since  for all *j*, we utilized a DFT code (Project Nayuki, https://www.nayuki.io/page/free-small-fft-in-multiple-languages) limited to data in the real-number domain. For such data both cosine and sine coefficients are real and  . Consequently,  =  and  =  for 1 ≤ *n* < *M*/2, and all coefficients are scaled by the number of sampled intensities *M*. Using these coefficients one can compute the original Bragg peak from the inverse DFT equation:

Here the summation is up to the Nyquist limit, *n*_NL_ = *M*/2.

In Fig. 14 ▸ the 111 peak profile obtained from the Patterson equation for a hypothetical 50 Å thick Au thin film using Cr *K*α radiation is shown, with Δ2θ_FR_ = 0.725 rad (approximately ∓8β) and δ2θ = 0.0008 rad; this yields *M* = 905 intensity values for the DFT analysis.

Computing the Fourier coefficients up to the Nyquist frequency *n*_NL_, we obtain 453 cosine and sine coefficients, which are plotted in Fig. 15 ▸(*a*). The sine coefficients  are negligible for all *n*, reflecting the symmetry of the peak profile. The cosine coefficients  vary linearly with *n* for 1 ≤ *n* ≤ 13 and decay to negligible values for *n* ≥ 14 [Fig. 15 ▸(*b*)]. The magnitude of the zeroth-order coefficient  , reflecting the integrated intensity of the peak profile over the measurement interval, is smaller than the absolute magnitudes of  for 7 ≤ *n*. As we mentioned above, this is a direct result of a DFT program limited to data in the real-number domain; when such programs are utilized in the analysis of experimental diffraction data for size and root-mean-square strain analysis, one can either exclude  from the set of Fourier cosine coefficients or multiply this value by two. In our work we exclude  . If we substitute all cosine and sine coefficients shown in Fig. 15 ▸(*a*) in the inverse DFT equation [equation (24)] to re-synthesize the 111 peak, we obtain an excellent fit with the peak profile computed using the Patterson equation (Fig. 14 ▸, red dots). In this frame the maximum intensity difference between the two traces is 4 × 10^−7^.

In our second approach we coded the following fast Fourier transform (FFT) equations in *MATLAB* (MathWorks, 2019 ▸):

(i) For Fourier decomposition,

(ii) For Fourier synthesis,

Equations (25*a*)[Disp-formula fd25a] and (25*b*)[Disp-formula fd25b] yield *M* cosine and sine coefficients, *M*/2 of which are independent. Thus, when applied to the Bragg peak shown in Fig. 14 ▸, we obtain 905 coefficients each for  and  [Fig. 16 ▸(*a*)]. As expected, with the exception of *n* = 0 and *n* = *M*/2, the magnitudes of the cosine coefficients obtained from equation (25*a*)[Disp-formula fd25a] are one half of those obtained from equation (23) [Fig. (16*b*) ▸]. This figure also intimates that  obtained from equation (25*a*)[Disp-formula fd25a] will exhibit the traditional monotonic decline (linear decline in the case of thin-film single crystals) from *n* = 0.

For this approach, *all* *M* coefficients computed from equations (25*a*)[Disp-formula fd25a] and (25*b*)[Disp-formula fd25b] are required to resynthesize the diffraction profile. Such general-purpose programs consume more resources than programs optimized for real numbers.

Finally, we tested the FFT algorithms in the *MATLAB* and *OriginPro* (OriginLab, 2021 ▸) software packages. Both programs use algorithms based on the *FFTW* subroutines (http://www.fftw.org) and yield ‘un-scaled’ Fourier coefficients. These must be scaled (normalized) by the number of data points (*i.e.* multiplied by 1/*M*) before they can be used in resynthesizing the Bragg peak profile. (While scaled coefficients must be used for synthesizing peak profiles, coefficients from all programs can be used directly as input for Fourier-based average size analysis, since  must be normalized (divided) by  or  for this purpose.)

### A2. Specification of frame range and number of data points

Equations (21*a*)–(26) show that, since Δ2θ_FR_ = *M*δ2θ, only two parameters of the DFT/FFT analysis can be independently specified to analyse Bragg peak profiles. In a recent article, de Avillez *et al.* (2018 ▸) investigated the optimization of these parameters for determining crystallite size distributions when confounding instrumental effects were present. Those authors did not extend the treatment to the simple Fourier-averaged sizes when instrumental effects were absent. Here, we use numerical analysis of the Bragg peak shown in Fig. 14 ▸ to investigate the influence of Δ2θ_FR_ and δ2θ on the accuracy of the average crystal dimension along the diffraction vector  .

To determine the optimum Δ2θ_FR_, δ2θ combination at minimum experiment and/or processing time, one can fix the frame range Δ2θ_FR_ and vary δ2θ, and then fix the optimum δ2θ obtained in the first step and change the frame range. We started by setting Δ2θ_FR_ = ∓8β, as shown in Fig. 14 ▸, and changing the step size. Relevant parameters are listed in Table 4 ▸. The average Fourier film thickness for each case (tabulated in the last column) was obtained from the intersection of the line fitted to its |*A_L_* | versus *L* data. These are shown in Figs. 17 ▸(*a*) and 17 ▸(*b*). For all reasonable step sizes the average film thickness is within 5% of the true film thickness. Even when the step size was not reasonable – there were only five points in the central peak for *M* = 33 – the average Fourier thickness of the film was within 5% of its true real-space value. The only effect of increasing the step size is the introduction of some nonlinearity for large *L*, *L* < *t*_f_. We conclude that 458 steps, corresponding to a step size 3.6% of the FWHM of the primary peak, are good enough for this ideal analysis.

For the second step of the optimization analysis we fixed the step size at 0.90° (0.00160 rad) and investigated the effect of frame range on  . Relevant details for this analysis are summarized in Table 5 ▸. We observe that decreasing the frame range dramatically curtails the number of Fourier coefficients  which yield column lengths in the linear range for the |*A_L_* | versus *L* plots (for *n* ≥ 1). Even when the entire central peak is sampled with 66 steps (second row of Table 5 ▸) we cannot perform the analysis. On the other hand, if the range includes just the central peak plus one pair of (bracketing) thickness fringes, the extrapolated  value is very accurate, even though the number of Fourier coefficients is quite small [Figs. 18 ▸(*a*) and 18 ▸(*b*)]. These observations imply that capturing most of the Bragg peak is quite important: for the same experimental duration it is better to use a larger step size (with appropriate counting time) over a broader frame range than to perform a fine-step scan over a narrower angular frame.

Finally, we investigated the effect of ‘zero padding’ on the analysis. In this procedure the frame range is artificially broadened by adding zero intensities to equidistant angular positions outside the measurement range. In our analyses we chose to extend the frames of simulations described by rows 1–4 in Table 5 ▸ to the full range shown in Fig. 14 ▸. Consequently, the Fourier transformation (DFT) acted on 458 intensity points in all cases. However, only a fraction of these intensity points centred on the primary peak were finite and ‘real’. The rest were ‘padding’.

‘Padded’ profiles containing the top 75 and 100% of the primary peak, with 40 and 66 original intensity values, *M*_o_ = 40 and 66, respectively, are shown in Figs. 19 ▸(*a*) and 19 ▸(*b*). The corresponding Fourier coefficients and the |*A_L_* | versus *L* plots (for *n* ≥ 1) obtained from these coefficients are shown in Fig. 20 ▸. For both cases, artificially expanding the frame range by tacking on zero intensities resulted in the recovery of 14 finite |*A_L_* | values for *L* ≤ *t*_f_. However, Figs. 20 ▸(*a*) and 20 ▸(*b*) show that padding introduces oscillations in the  values. These oscillations decay with increasing ‘real’ frame range and are due to the artefacts introduced into the peak profiles by the padding. We also observe both oscillations and curvature in the |*A_L_* | versus *L* plots (for *n* ≥ 1). These also diminished with increasing true frame range, vanishing completely for adequate sampling: for *M*_o_ ≥ 264, the |*A_L_* | versus *L* plots with 454 real intensity points, and with 264 or more real intensity points padded to 454 total points, were coincident.

In Table 6 ▸ we list the average film thicknesses  obtained from the |*A_L_* | versus *L* plots (for *n* ≥ 1) by fitting the linear central ranges. The computed values are sufficiently accurate for most structural characterization applications. We conclude that Fourier analysis, properly implemented, is quite robust for the determination of average domain size in single-crystal (or mono-disperse) polycrystalline samples with no strain broadening effects, even when padding is used. On the other hand, ascribing nonlinearities in Fourier coefficient plots and their extension to structural features of the sample appears to be non-trivial. Such effects can also be caused by the type of algorithm used for computing the raw Fourier coefficients,  and  , or be due to asymmetries in the peak shape caused by frame range selection, padding or other non-sample-related reasons.
